# 

**Published:** 1889-04

**Authors:** 


### BOOKS AND PAMPHLETS RECEIVED.

"Swine Plague and Hog Cholera Critically Considered," by Frank S. Billings, Director Patho-Biological Laboratory, of the State University of Nebraska.

"Transactions of the American Dermatological Association—Twelfth Annual Meeting." Boston, 1888.

"Massachusetts Agricultural College Bulletin"—"Tuberculosis," by Chas. H. Fernald. No. 3, 1889.

"Bulletin Agricultural Station, Cornell University." No. 14, 1888.

"The Cortical Localization of the Cutaneous Sensations," by Charles L. Dana, M.D. Reprinted from the *Jour. Nervous and Mental Diseases.* October, 1888.

"Note on Rumbold's Method of Treatment of Catarrhal Inflammations of the Upper Air Passages," by Ely McClellan, M.D., U.S.A. Reprinted from the *Jour. Amer. Med. Assoc.*, Chicago, 1889.

"Report on the Proceedings of the U. S. Expedition to Lady Franklin's Bay," by A. W. Greely, U.S.A. Vol. II. Washington, 1888.

"Contributions to the Comparative Osteology of Artic and Sub-Artic Water Birds," by R. W. Shufeldt, M.D. Part II. Reprinted from *Jour. Anat. and Physiology*, vol. xxiii.

"On the Affinities of Apriza virgata, and Further Studies on Grammecolepsis brachiusculus," by R. W. Shufeldt, M.D. Reprinted from *Jour. Morphology*, vol. ii, No. 2.

"The Navajo Tanner," by R. W. Shufeldt, M.D. Reprinted from "Proc. U. S. Nat. Museum," 1888.

"Sitzungsberichte der Mathematisch Physikalischen Classe der K. b. Akademie der Wissenschaften zu Munchen, 1880–1887." 1888, Heft 1.

Publications of the Ksl. Leop. Carol. Deutschen Akademie der Naturforscher.

"Uber Resectionen und Amputationen," von Dr. J. F. Heyfelder.

"Uber die Entwickelung und den Bau des Saugethierzahns," von Dr. A. Hannover.

"Uber den Bau des Gehirns der Fische in Beziehung auf eine darauf gegrundete Eintheilung dieser Thierklasse," von Dr. F. J. C. Mayer.

"Uber den Bau der Nase der Antilope Saiga," von Dr. L. Glitsch.

"Zur Kenntniss der Zahnformel fur die Gattung Sus," von R. Hensel.

"Photogramme zur Ontogenie der Vogel," von C. Kupffer und B. Benecke.

"Uber das os intermaxillare des Menschen und die Anatomie der Hasencharte und des Wolfsrachens," von Dr. T. Kolliker.

"Die Mallophagen mit besonderer Berucksichtung der, von Dr. Meyer gesammelten Arten" von Dr. O. Taschenberg.

"Gewichtsbestimmungen zur Entwickelung des Muskelsystems und des Skelettes beim Menschen," von F. W. Theile.

"Beitrage zur vergleichenden Anatomie und Entwickelungsgeschichte des unteren Kehlkopfes der Vogel," von. L. Wunderlich.

"Mikrographe der Mitteldarmdruse der Mollusken," von Dr. J. Frenzel.

"Die Entwickelungs und Lebensgeschichte von *Chaitophorus*," von H. F. Kessler.

"Untersuchungen uber den Bau der Speicheldrusen und des dazu gehorenden Nervenapparats von Blatta," von B. Hofer.

"Beitrag zur Kenntniss des Pferdegebisses mit Rucksicht auf die fossilen Equiden von Maragha in Persien," von Dr. M. Wilckens.

AMIENS.—*Bulletin Societe Linneenne du Nord de la France.*

AUGUSBURG.—*Wochenschrift fur Thierheilkunde und Viehzucht.*

BALTIMORE.—*Johns Hopkins University Circulars.*

BATAVIA.—*Veeartsenijkundige Bladen voor Nederlandsch-Indie.* Deel iii, Afl. 3–4.

BERLIN.—*Archiv fur Wissenschaftliche und Praktische Thierheilkunde.* Bd. xv, Heft 1–2.—*Sitzungs-Berichte der Gesellschaft Naturforschender Freunde.* Jahr 1888. Contains "Forms of the Canine Teeth of the Various Species of Wild Swine," "On the Skeleton of the *Bos primigenius*," "Influence of Domestication in Relation to the Size of Animals, Noting Especially the Difference of Size Between the Wild and Tame Yak—*Poephagus grunniens.*"

BOMBAY.—*Journal of the Natural History Society.* Vol. iii, No. 4. Contains "Notes on the Dambhur Deer," by R. Gilbert; "The Indian and Burmese Scorpions," by E. W. Oates.

BONN.—*Verhandlungen des Natur-historischen Vereines.* Folge v, Half 2, 1888.

BRISBANE.—*Proceedings of the Royal Society of Queensland.* Vols. 4–5, Parts 3–4. Contains "Notes on Bovine Pleuro-Pneumonia in Queensland," by Edward Palmer; "On an Extinct Mammal of a Genus apparently new," by C. W. de Vis; "On an Unrecorded Habit of White Ants," by Henry Tryon; "Observations on Cataract," by E. J. Bennett; "Australian Ancestry of the Crown Pigeon of New Guinea," by C. W. de Vis.

BRUSSELS.—*Annales de Medecine Veterinaire.*—*Bulletin de l'Academie Royal de Medecine de Belgique*, 1880—1889. Part i.

BUENOS AIRES.—*Anales de Museo Nacional.* Entrage xv. Contains "Description of an Expedition to Patagonia, with List of Fauna," by C. V. Burmeister.

CAPE ROUGE.—*Le Naturaliste Canadiene.*

CHICAGO.—*The Weekly Drovers' Journal.—National Live Stock Journal.*

CINCINNATI.—*Journal Society of Natural History.* Contains "Contribution to the Ichthyology of Ohio," by J. A. Henshall; "Riverside Skull," by A. J. Howe.

COPENHAGEN.—*Tidsskrift for Veterinaerer.*

DETROIT.—*The Microscope.*

EDINBURGH.—*Journal of Comparative Pathology and Therapeutics.*

FRANKFORT.—*Der Zoologische Garten.*

GHENT.—*Annales et Bulletin de la Societe de Medecine de Gand.*

GRATZ.—*Mittheilungen des Naturwissenschaftlichen Vereins fur Steiermark.* 1888. Contains "Hymenoptera of Styria," by E. Hoffer; "List of Birds at Furt Pond, 1887," by P. B. Hauf; "Phylloxera on Grape Vines," by G. Wilhelm; "List of West Palæartic Snakes," by A. von Mojsisovics.

HALLE.—*Leopoldina.* Vols. xvi–xxii.

HARLEM.—*Archives Neerlandaises des Sciences exactes et Naturelles.* Tome xxii–xxiii, Liv. 1 Contains "A Case of Polydactyla," by C. H. H. Spronck; "Contributions to Vegetable Pathology," by J. H. Wakker.

HAVANA.—*Revista de Ciencias Medicas.*

HERMANNSTADT.—*Verhandlungen und Mittheilungen des Siebenburgischen Vereins fur Naturwissenschaften*, xxxviii, Jahr. 1888. Contains "The Vertebral Fauna of Transylvania," by E. A. Bielz; "The Duties of Professors of Hygiene in the Public Schools," by H. Sussmann.

JENA.—*Jenaische Zeitschrift fur Medizinisch-Naturwissenschaftlichen Gesellschaft.* Contains "Contribution to the Development and Anatomy of the Sexual Apparatus of Limulus," by J. Klots; "A Contribution to the Anatomy and Ontology of the Nematoids," by N. A. Cobb.

KARLSRUHE.—*Thierarzliche Mitthielungen.*

KNOXVILLE.—*Agricultural Science.*

LONDON.—*The Veterinary Journal.—The Veterinarian.—The Lancet.*

LYONS.—*Journal de Medecine Veterinaire et de Zootechnie.*

MADRAS.—*Quarterly Journal of Veterinary Science in India.*

MADRID.—*Gaceta Medico-Veterinaria.*

MILAN.—*La Clinica Veterinaria.*

MONTREAL.—*Canadian Record of Science.* Vol. iii, No. 5. Contains "The Influence of the Nervous System on Cell Life," by T. Wesley Mills, M.D.

NEW YORK.—*Medical Analectic.—New York Medical Journal.—Scientific American.—Forest and Stream.—American Veterinary Review.—Spirit of the Times.—The Sanitarian.*

OTTAWA.—*Ottawa Naturalist.*

PALERMO.—*Il Naturlista Siciliano.* Anno viii, Nos. 3–5. Contains "The Lepidoptera Fauna of Sicily," "On two Species of Locusts new to Sicily," "A Study on the Belgian Species of *Psithyrus*," "On the Humor Secreted from the Timarcha."

PARIS.—*Le Progress Medical.—Recueil de Medecine Veterinaire.—Bulletin de la Societe Zoologique de France.* Tome xiii, Nos. 9–10; Tome xiv, No. 1. Contains "On the Transmission of Color in the Batrachians," by G. Heron Royer; "Lamellibranchi Without Gills," by W. H. Dall; "The Parasites of the Chabin," by A. Railliet; "The Fetus of the Needle Fish," by H. E. Sauvage; "On the Reproduction of the Foraminifer," by C. Schlumberger.

PAVIA.—*Bollettino Scientifico.* Contains "History of the Cabinet of Human Anatomy in the University of Pavia," by G. Zoja.

PISA.—*Giornale di Anat. Fisiol. e Patol. Degli Animali.*

RED WING.—*Public Health in Minnesota.*

SHANGHAI.—*Journal of the China Branch of the Royal Asiatic Society.* Vol. xxii, No. 6; vol. xxiii, No. 1.

STOCKHOLM.—*Tidskrift for Veterinar-Medicin.*

ST. PETERSBURGH.—*Archives of Veterinary Science.*

STUTTGART.—*Repertorium der Thierheilkunde.*

SYDNEY.—*Proceedings of the Linnean Society of New South Wales.* Vol. iii, Part 3. Contains "Insects of King's Sound and Vicinity," by W. Macleay; "Catalogue of Coleoptera of New Guinea," by Geo. Masters; "Malaysian Mollusca," by Rev. J. E. Tenison-Woods; "Diptera of Australia," by F. A. A. Skuse; "Post-Tertiary Avifauna of Queensland," by C. W. de Vis.

TOULOUSE.—*Revue Veterinaire.*

TURIN.—*Bolletino di Musee de Zoologie ed Anatomia Comparata.* Vol. iii, Nos. 49–52. Contains "Monograph of the Italian Ophidea," by L. Camerano.

UDINE.—*Giornale di Veterinaria Militaire.*

VALENCIA.—*La Cronica Medica.*

VIENNA.—*Œsterreichische Monatschrift fur Thierheilkunde.—Mittheilungen des Ornithologischen Vereins.—Annalen des K. K. Naturhistorischen Hofmuseums.* Bd. iii, No. 3. Contains "The Honey Bee Collection of the Hofmuseum," "Flora of the Stewart-atolls in the Pacific Ocean," "Foraminifer in the Chalk of South Austria," "The Flora of New Caledonia."—*Verhandlungen der K. K. Zoologisch-Botanischen Gesellschaft.* Bd. xxxviii, Quarts. 3–4. Contains "A Study of the Æolidia," by R. Bergh; "Hymenoptera Fauna of the Tyrols," by F. F. Kohl; "Austrian Tipulidæ," by E. Bergroth.

